# Formative Assessment: Design of a Web-Connected Sedentary Behavior Intervention for Females

**DOI:** 10.2196/humanfactors.7670

**Published:** 2017-10-20

**Authors:** Amber W Kinsey, Matthew Whipple, Lauren Reid, Olivia Affuso

**Affiliations:** ^1^ Nutrition Obesity Research Center University of Alabama at Birmingham Birmingham, AL United States; ^2^ Northrop Grumman Corporation Atlanta, GA United States; ^3^ Department of Epidemiology and Biostatistics University of South Carolina Columbia, SC United States; ^4^ Department of Epidemiology University of Alabama at Birmingham Birmingham, AL United States

**Keywords:** health promotion, qualitative research, sedentary lifestyle, motivation

## Abstract

**Background:**

Sedentary behavior (SB) is a significant risk factor for heart disease, diabetes, obesity, and early mortality, particularly among women, and the health consequences associated with SB are independent of physical activity status. Interventions utilizing wearable technologies can improve SB, but their effectiveness is influenced by individual preferences, device engagement strategies, and technological features, which may affect user compliance. Gathering a priori insight from target populations on their preferences for program tools and strategies may assist researchers in identifying effective methods to improve the efficacy of SB interventions.

**Objective:**

The objective of this study was to (1) explore the likeability (likes and dislikes) and usability (engagement intentions and navigation) of a wearable device (Movband) and its accompanying website (dashboard), (2) examine social incentive preferences (teammates), and (3) assess the feasibility (participants’ experiences during an activity-monitoring period) of these tools for use in an intervention to reduce SB in girls and women.

**Methods:**

A total of 9 girls (mean age: 8.9 years, standard deviation [SD] 1.1 years) and 11 college-aged women (mean age: 22.6 years, SD 3.2 years) participated in this study. Separate focus groups were held for girls and women, and all participants attended one before and the other following a 7-day activity-monitoring period. During the focus groups, participants were prompted with questions to address the study aims, and the nominal group technique was used to compile lists of group-specific preferences for the activity-monitoring system. The top three ranking likes and dislikes were reverse coded to determine likeability.

**Results:**

The top-ranking responses for the girls and women were the following: visual display of movements and ease of navigation (dashboard like), boring to look at and no calorie-tracking function (dashboard dislike), backlight and long battery life (Movband like), and color and not waterproof (tied for girls) and vertical time display (Movband dislike). Additionally, participants identified several aesthetic preferences and functional limitations. At the second focus group visit, the majority of the participants self-reported less SB during the previous week. Objective data from the activity-monitoring period revealed that the average steps per day for girls and women were 12,373.4 (SD 2617.6) and 8515.8 (SD 3076.7), respectively.

**Conclusions:**

These results suggest that the girls and women liked many features of the Movband and dashboard. However, several dislikes were mentioned, which may negatively influence compliance and the effectiveness of the activity-monitoring system and require improvements before using in an SB intervention.

## Introduction

A lifestyle characterized by significant periods of inactivity or sedentary behavior (SB) [1] represents a major risk factor for heart disease, diabetes, obesity, and early mortality, particularly among women [2-5]. Interventions to reduce sedentariness in girls and young women are not well established [6,7], but those exclusively targeting SB, as opposed to in combination with physical activity, appear to produce the greatest improvements [8]. More importantly, whereas a high prevalence of SB exists [9,10], particularly in those living in the southern region of the United States [11,12], the health consequences associated with SB are independent of physical activity status [13]. Thus, the need for effective intervention strategies to improve SB in this population is apparent.

### Technology for Activity Promotion

Using technologies such as the Internet and wearable devices is efficacious in promoting activity-related behavior changes [14,15]. Electronic activity-monitoring systems, consisting of a wearable device and accompanying website and/or mobile app, collect objective measures of lifestyle activity and provide feedback beyond the display of basic activity count information from a device alone to facilitate self-monitoring [16]. Studies utilizing these activity-monitoring systems to increase physical activity in youth [17] and adults [16] have shown promise; however, less is known about their influence on SB [15,16,18]. Wearable technology in itself can improve sedentariness by increasing the user’s awareness of the behavior [19], but the effectiveness of emerging technologies is highly influenced by individual preferences, device engagement strategies, and technological features [20,21], all of which can affect compliance to device use and the achievement of activity goals.

### Appealing to the Target Population

Developing programs that appeal to target populations may improve the efficacy of interventions utilizing wearable technology by maximizing participants’ engagement and compliance in the program. One concept that is particularly relevant to health promotion efforts targeting specific groups is the marketing mix, which involves the integration of four elements (ie, product, price, place, and promotion; the 4Ps), to satisfy consumer needs and wants with the goal of facilitating behavior changes [22]. Here, we focus only on the product, which has three forms: core (ie, the underlying benefit to the consumer), tangible (ie, the physical product), and augmented product (ie, additional features influencing long-term compliance) [22]. Related to improving sedentariness, the products are the reduced health risks associated with less SB (*core product*) and the strategic facilitators used to support these changes (ie, wearable devices and user engagement strategies; *tangible and augmented products*). An understanding of girls and young women’s preferences for the tangible and augmented intervention components may enhance the efficacy of these tools to produce the desired behavior changes.

### Social Incentives

Enhancing motivation for behavior change is commonly achieved through the use of incentives (*augmented products*), which can be monetary or social in nature. Although financial incentives can encourage individuals to make changes in behavior [23], they may also undermine the potential increases in enjoyment for positive health behavior changes [24]. In contrast, social incentives (eg, partners/teams, competition, and altruism) have been associated with enjoyment and improvements in activity patterns [25-28]. Partner-based programs, in particular, have been associated with motivation, social support, and accountability for physical activity–related changes [26-28], whereas the use of this strategy to improve SB is unknown.

### Purpose

On the basis of the need to improve SB, studies supporting the efficacy of wearable technologies to improve activity patterns and the evidence demonstrating that preferences and social incentives may influence health behaviors, the aims of this study were to use formative assessments (1) to gain knowledge on the likeability and usability of an activity-monitoring system in girls and young women, (2) to examine social incentive preferences, and (3) to assess the feasibility of these tools for use in an intervention to reduce SB in this population.

## Methods

### Participants

Healthy girls (aged 8-11 years) and young women (aged 19-30 years) were recruited from Birmingham, Alabama, through flyers, print and Web-based ads, and through word of mouth. Prospective participants were screened via phone or email to ascertain eligibility. Specifically, prospective participants’ eligibility was determined by their responses to the following questions:

1. Do you (does your child) have any medical conditions that would prevent you (them) from participating in physical activities?
2. Have you used any physical activity–monitoring devices in the past 3 months?
3. Do you (does your child) have an allergy to latex?
4. What is your (your child’s) current activity level?

For the adults, the different activity parameters were classified by their response to the following statement: a daily profession where literally no exercise is done and most of the time is spent sitting in a chair (no exercise); some time in a day is spent moving from place to place, spending some time at a desk or in a chair (some exercise); most of the day is spent working as a skilled labor (moderate exercise); or most of the day is spent doing some physical work or involves daily exercise for most of the day (athletic). When screening parents or legal guardians (herein referred to as parents) of prospective minors, the content was adjusted to include terminology specific to children (eg, asking about physical activity habits, sports and recreation involvement). Those who self-reported no medical conditions that would prevent them from engaging in physical activity, had not worn a physical activity monitor in the past 3 months, had no allergies to latex, and indicated that they had a sedentary or moderately active lifestyle were included in the study. Age, height, weight, and race/ethnicity were also self-reported by the women and parents. The body mass index (BMI) was calculated for women (weight in kg/height in meters squared), whereas the BMI percentile and z-scores were determined for the girls [29]. Written informed consent from the women and parents, in addition to child assent, was obtained before their study involvement. The institutional review board at the University of Alabama at Birmingham approved this study (X150120004).

### Design

Each participant attended two focus groups, one before and the other following a 7-day activity-monitoring period. Recognizing that the age-related preferences are likely to exist, separate focus groups were held for girls and women. These focus groups were structured to elicit information on the likeability and usability of an electronic activity–monitoring system that consisted of a wearable device (Movband, Model 2, DHS Group, Houston, TX, USA) and its accompanying website (hereafter referred to as the dashboard). Likeability was evaluated by participants’ perception (ie, likes and dislikes) of the dashboard and activity monitor. Usability was evaluated by participants’ engagement intentions, navigation of the system, and feature and functionality preferences. Social incentive preferences were explored by assessing participants’ interest and age preferences for a teammate. Feasibility was evaluated by examining participants’ experiences during the activity-monitoring period (ie, device failure, forgetting to put it back on after showering, etc). To accomplish these goals, the nominal group technique, a structured variation of a small group discussion that allows for full group participation and results in a set of prioritized responses, was employed as described elsewhere [30]. However, individual responses were verbalized to the entire group, as opposed to individually written [30], and subsequently recorded on a large easel pad by the facilitator. Thereafter, participants ranked their top three responses from the compiled list (see *Focus Groups* section).

### Focus Groups

**Figure 1:**
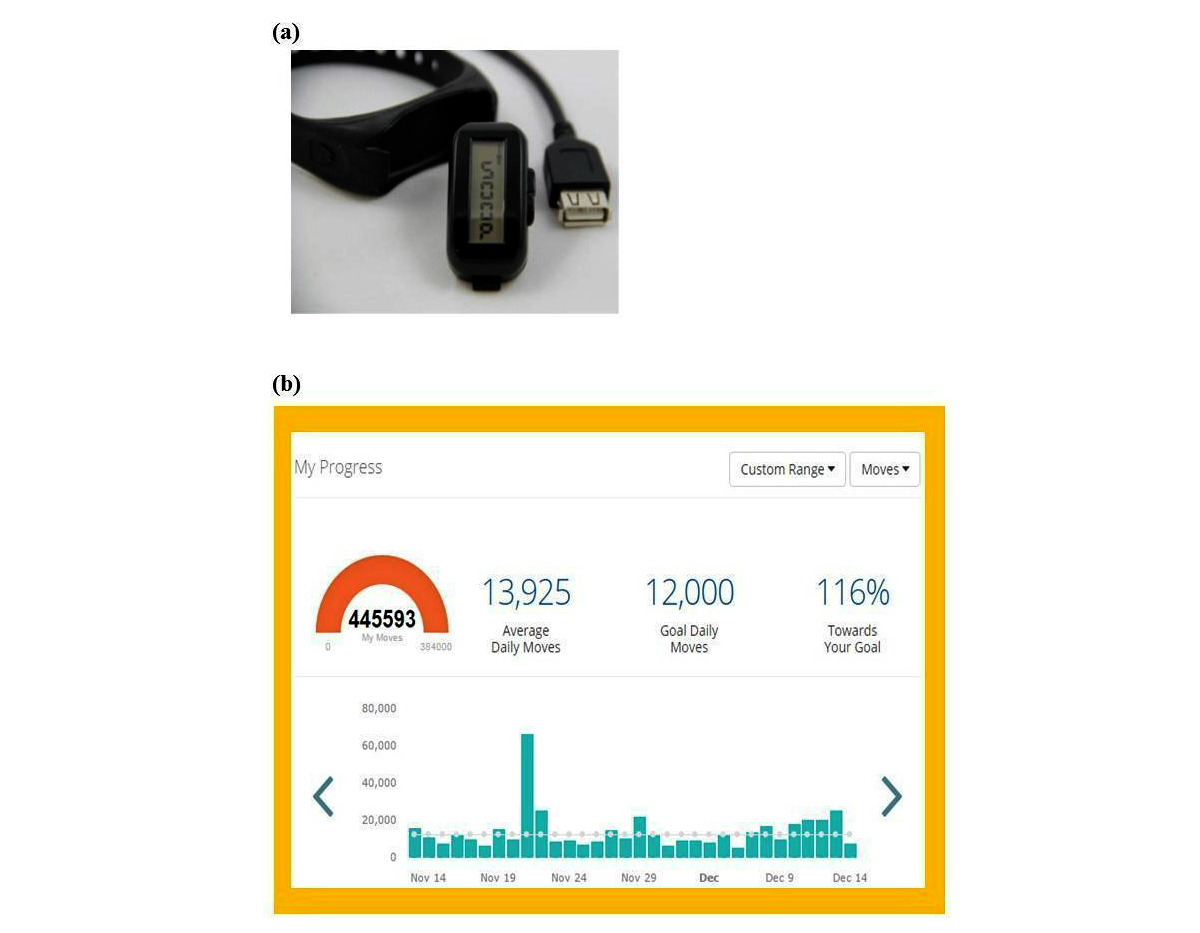
Movband and Dashboard. The dashboard allows users to view graphical activity data (ie, moves, steps, and miles) over a custom time frame (ie, days, weeks, months, custom), daily activity goals, and one’s progress toward reaching the daily goals.

A summary of the dashboard and Movband features are displayed in Textbox 1. During the first focus group, participants sat around an oval table in groups of 2 or 3, sharing a computer that displayed the dashboard containing sample activity data from a user (OA). The dashboard is a three-part platform and, upon log-in, the default platform provided users with graphical displays of activity (ie, moves [a measure of physical activity], steps, and miles) that were viewable over a custom time frame (ie, days, weeks, months, and custom). The dashboard display included preset activity goals (ie, 12,000 moves, 10,000 steps, or 4 miles) and an indicator of one’s progress toward their daily goal (Figure 1). Participants were given a 5-min observation period to explore the dashboard features and, thereafter, were presented with a series of questions (Table 1). Where appropriate, as indicated in Table 1, participants were asked to individually rank their top three responses from the list of compiled responses and record them on the small piece of paper provided.

Participants were then given a 5-min observation period to explore a black Movband device (Figure 1). The Movband is a wrist-worn accelerometer that syncs with the dashboard via Universal Serial Bus (USB). On the device, users can view their daily moves and mileage; to view steps, users must access the dashboard. By design, the device resets the moves to zero each night at midnight, whereas the miles accumulate over time. Movband’s proprietary algorithm takes into account pace, movement intensity, and stride length to calculate moves and miles. Stride length is determined by age, sex, and height. Although users may accumulate many moves in low-intensity activity, greater mileage is the result of fast-paced, high-intensity activities. Preliminary evidence suggests that the Movband is valid for children [31,32] and has been used for adults [33]. Following the Movband observation period, the participants were asked related questions and individually ranked their responses where appropriate (Table 1). Lastly, participants were asked about their willingness to have a teammate (Table 1) to explore their interest and age preferences for a partner.

Following the first focus group, participants were compensated US $10 and asked to wear the Movband at all times, with the exception of bathing and showering, until their second focus group visit 1 week later. This activity-monitoring period was used to obtain objective activity measures. During their second visit, participants synced their device with the dashboard, observed their activity from the previous week, and returned their Movband. Participants responded to questions, which included a subjective measure of SB and problems experienced and ranked their responses accordingly (Table 1). Following this visit, participants were compensated an additional US $10.

### Analyses

Summary statistics were calculated for the participant characteristics. Where appropriate, rankings were tabulated for each item and the top three responses were reverse coded as follows: first, 3 points; second, 2 points; third, 1 point. Responses with tied rankings were also reported. All unique responses from the participants are displayed in the Multimedia Appendix 1. Quantitative data are presented as the mean (standard deviation; SD).

**Features of the dashboard and Movband.**

Dashboard

- Graphical displays of moves, miles, and steps that can be viewed over a custom time frame
- Personalized goal-setting capabilities and progress toward goal indicators
- Group-based dashboard
- Compatible with third party activity trackers and apps (eg, Fitbit, Garmin, Jawbone, and MapMyFitness)
- Front-end system for user interaction
- Admin Center for researchers to create groups and challenges, set goals, and communicate via email with group members

Mobile phone app available

Movband 2

- Wrist-worn accelerometer
- Wristband available in multiple colors
- Consumer purchase price of US $30
- Visual display of moves and miles on device
- Tracks moves (a measure of physical activity), miles, and steps
- Vertical time display
- Universal Serial Bus (USB) syncing
- Rechargeable battery
- 30-day battery life

**Table 1:** Focus group questions.

Questions		Focus group visit^a^	Ranked
**Questions for Dashboard**			
	What do you like about this website?	1	Yes
	How often would you log-in to the website to see your movements?	1	-
	What is your least favorite feature on this website?	1, 2	Yes
	What is your favorite feature on the website?	2	Yes
	What problems did you experience while using the website?	2	-
**Questions for Movband**			
	What do you like about the Movband?	1	Yes
	What do you not like about the Movband?	1	Yes
	After using the Movband for a week, what do you like about it now?	2	Yes
	What do you not like about it now?	2	-
	Do you feel like you were less sedentary in the past week? If so, what did you do differently?	2	Yes
**Question for teams**			
	How do you feel about having a teammate who is younger (for the women) /older (for the girls) than you?	1, 2	-

^a^Each participant attended two focus groups, one before and the other following a 7-day activity-monitoring period. Numbers indicate visit during which the question was asked.

## Results

### Participants

A total of 29 women and 12 girls were screened for eligibility (recruitment source: flyers [n=6], Web-based ads [n=29], and word of mouth [n=6]). Of this sample, 14 women and 10 girls were deemed eligible, and 11 women and 9 girls participated in this study. Participants’ characteristics are provided in Table 2. Of the total participants 56% girls (5/9) and 72% women (8/11) in our sample were classified as overweight or (ie, average BMI ≥25 or ≥30 kg/m^2^[women]; BMI percentile ≥85th or ≥95th [girls]). Furthermore, 67% girls (6/9) and 55% women (6/11) were ethnic minorities. Parents reported most of the girls (67%, 6/9) to be moderately active, whereas most of the women (82%) engaged in some activity (Table 2).

### Likeability

Likeability was evaluated by participants’ perception of the dashboard and Movband monitor. For the dashboard, the highest-ranking responses (of 23 unique responses; see Multimedia Appendix 1) for overall likeable features reported by girls were the visual display of movements (score of 15; visit 2) and the ability to recall activity from the past (score of 13; visit 1). The ability to store steps, moves, and miles (visit 1) and daily measurements and tracking over time (visit 1) were tied in rankings with a score of 8 (Table 3). Goal attainment was another feature liked by the girls and cited during both focus group visits. For the women, the highest-ranking likeable dashboard features (of 24 unique responses) were its ease of navigation (score of 21; visit 1), goal attainment (score of 17; visit 2), and incentives and prizes (score of 13; visit 1). The women also cited the hour-by-hour breakdown and the dashboard interface as favored features. Regarding the dashboard dislikes, the highest-ranking response (of 13 unique responses) cited by the girls was boring to look at (score of 16; visit 1), followed by worries of forgetting their log-in password (score of 14; visit 1) and the color (score of 9; visit 2). In contrast, the highest-ranking dislike (of 21 unique responses) for the women was the inability to connect to a calorie tracker (score of 12; visit 2), followed by the inability to personalize goals (score of 11; visit 1) and the inability to track heart rate, which were tied in rankings (score of 11; visit 1).

The backlight feature of the Movband (score of 13; visit 2) was ranked the highest (of 20 unique responses) among the girls. Other favorable features included its ability to count steps (score of 9; visit 1), the ease of use (score of 8; visit 1), and mile accumulation feature (score of 8; visit 2). The women ranked the long battery life (score of 15; visit 1) the highest (of 28 unique responses), followed by price (score of 13; visit 1) and the time display (score of 12; visit 2). Top ranking dislikes cited by the girls (of 28 unique responses) were the color (score of 11; visit 1), not waterproof (score of 11; visit 2), as well as not being pretty and the vertical time display (tied in rankings with a score of 10; visit 1,). Similarly, the women cited vertical time display (score of 13; visit 1) and not waterproof (score of 12; visit 1) and tied in rankings with a score of 11, the rectangular shape (visit 1) and lack of date feature (visit 2), as their top-ranking dislikes (of 29 unique responses).

### Usability

Usability was evaluated by participants’ engagement intentions, navigation of the system, and feature and functionality preferences. Regarding website engagement (ie, *How often would you log into the website?*—Table 1), the girls responded with a log-in duration (ie, *2 or 4 hours a day*) or mentioned frequency (ie, every day) (*at lunch, everyday*, *every single day*, *every day*, *every afternoon*, and *morning and night*). Other responses were weekends (*Saturday morning, afternoon, and evening*) or outside school time (*days I’m off from school*). Women indicated that they would engage with the website daily (using an app), weekly, multiple times per week, or monthly. The range of system dislikes and functional limitations and preferences identified by our participants suggest that women were more thorough than the girls during the observation periods as some of their preferences (Table 4 and Multimedia Appendix 1) revealed that their navigation through the system went beyond that of the “default platform” displayed in Figure 1. Likewise, some preferred features described by our participants are readily available in the dashboard but went unnoticed (eg, personalized goal setting; Textbox 1). There were no website-related problems mentioned by the girls. However, two general issues were mentioned by the women: problems syncing their devices after the activity-monitoring period (because of a company upgrade that we were unaware of) and the presence of an error message despite “fixing” the error. One woman noted that she was not technologically savvy and may need a cheat sheet to navigate the website.

**Table 2:** Participant characteristics.

Characteristics		Girls (N=90)	Women (N=11)
Age in years, mean (SD^a^)		8.9 (1.1)	22.6 (3.2)
Height in inches, mean (SD)		54.4 (4.0)	65.5 (2.9)
Weight in pounds, mean (SD)		76.7 (21.9)	167.1 (27.4)
BMI^b^, kg/m^2^, mean (SD)		-	27.4 (3.8)
BMI, z-score, mean (SD)		−0.05 (2.2)	-
BMI, percentile, mean (SD)		53.5 (43.9)	-
**Weight status classification (n)**			
	Underweight	1	-
	Normal weight	3	3
	Overweight	2	6
	Obese	3	2
**Race/ethnicity (n)**			
	African American	4	5
	Non-Hispanic white	3	5
	Hispanic	1	0
	Asian	1	1
**Activity level**			
	None	-	1
	Some	3	9
	Moderate	6	1

^a^SD: standard deviation.

^b^BMI: body mass index.

**Table 3:** Summary of focus group responses for the dashboard and Movband for girls.

Responses for girls		Focus group visit		Score	
**Dashboard likes**					
	Ability to recall activity from the past		1		13
	Ability to store steps, moves and miles^a^		1		8
	Daily measurements^a^		1		8
	Tracking activity over time^a^		1		8
	Progression toward goal attainment		1		5
	Visual display of movements		2		15
	Ability to see goal attainment		2		8
	Same as the previous week^b^		2		7
	Display of total steps^b^		2		7
**Dashboard dislikes**					
	Boring to look at		1		16
	If you forget your password, you can’t get in		1		14
	Nothing		1		8
	Boring to look at		2		16
	Color of dashboard		2		9
	Does not display entire total		2		8
**Movband likes**					
	Ability to count steps		1		9
	Ease of use		1		8
	Comfortable		1		5
	Ability of screen to light up		2		13
	Miles do not reset every night		2		8
	Tells the time		2		7
**Movband dislikes**					
	Color		1		11
	Not pretty^c^		1		10
	Vertical time display^c^		1		10
	Nothing^d^		1		7
	Flat and uncomfortable on wrist^d^		1		7
	Not waterproof		2		11
	Uncomfortable in general and to sleep with		2		9
	Sometimes uncomfortable		2		6

^a,b,c,d^Matching letters indicate responses with tied rankings by visit.

^e^Top three responses from a compiled response list were individually rank and reverse coded as follows: first, 3 points; second, 2 points; third, 1 point (range of possible scores: 1-33).

**Table 4:** Summary of focus group responses for the dashboard and Movband for women.

Responses for women		Focus group visit	Score
**Dashboard likes**			
	Ease of navigation	1	21
	Incentives and prizes	1	13
	No advertisements	1	8
	Goal attainment	2	17
	Hour-by-hour breakdown	2	12
	The dashboard	2	10
**Dashboard dislikes**			
	Inability to personalize daily goals^a^	1	11
	Does not allow tracking of heart rate^a^	1	11
	Cannot compare activity with weight loss	1	10
	No Bluetooth capability	1	7
	Not connected to calorie tracker app	2	12
	Inability to set daily goals	2	9
	Inability to track weight loss	2	6
**Movband likes**			
	Long battery life	1	15
	Price	1	13
	Narrow wristband	1	7
	Time display	2	12
	No need to charge it	2	10
	Good feedback on moves and steps	2	9
**Movband dislikes**			
	Vertical time display	1	13
	Not waterproof	1	12
	Rectangular shape	1	11
	Watch does not show the date	2	11
	Unable to wear monitor other than on the wrist	2	9
	Need for smaller wristband	2	5

^a^Matching letters indicate responses with tied rankings by visit.

^b^Top three responses from a compiled response list were individually rank and reverse coded as follows: first, 3 points; second, 2 points; third, 1 point (range of possible scores: 1-27).

### Social Incentive Preferences

Social incentive preferences were explored through the assessment of participants’ interest and age preferences for a teammate. At the first visit, the majority of the girls’ responses were in support of having an older teammate, citing the opportunity to meet new people and having a friend with whom they could discuss their activity. Only two responses indicated that a teammate may not be preferred (“horrible” and “I don’t like it but I just go with the flow”). At visit 2, all of the girls were in favor of having a teammate and mentioned their excitement and the benefits of the teammate, which included the potential teammates’ maturity level, friendship, and knowledge. During both focus group visits, the women expressed their interest to have a younger teammate for the following reasons: competition, motivation, accountability, role model, and support. Two responses from the women also revealed that their teammates’ current activity level was more important than their age. However, some concerns regarding the awkwardness with not knowing their teammate (visit 1) and a preference to not interact with a teammate and “just wear it [the Movband] and forget about it” (visit 2) were mentioned. Other responses indicated that teammate sex concordance (visit 1), having goals (visit 1), and the ability to set up a private chat between teammates would be ideal (visit 1).

### Feasibility

**Figure 2:**
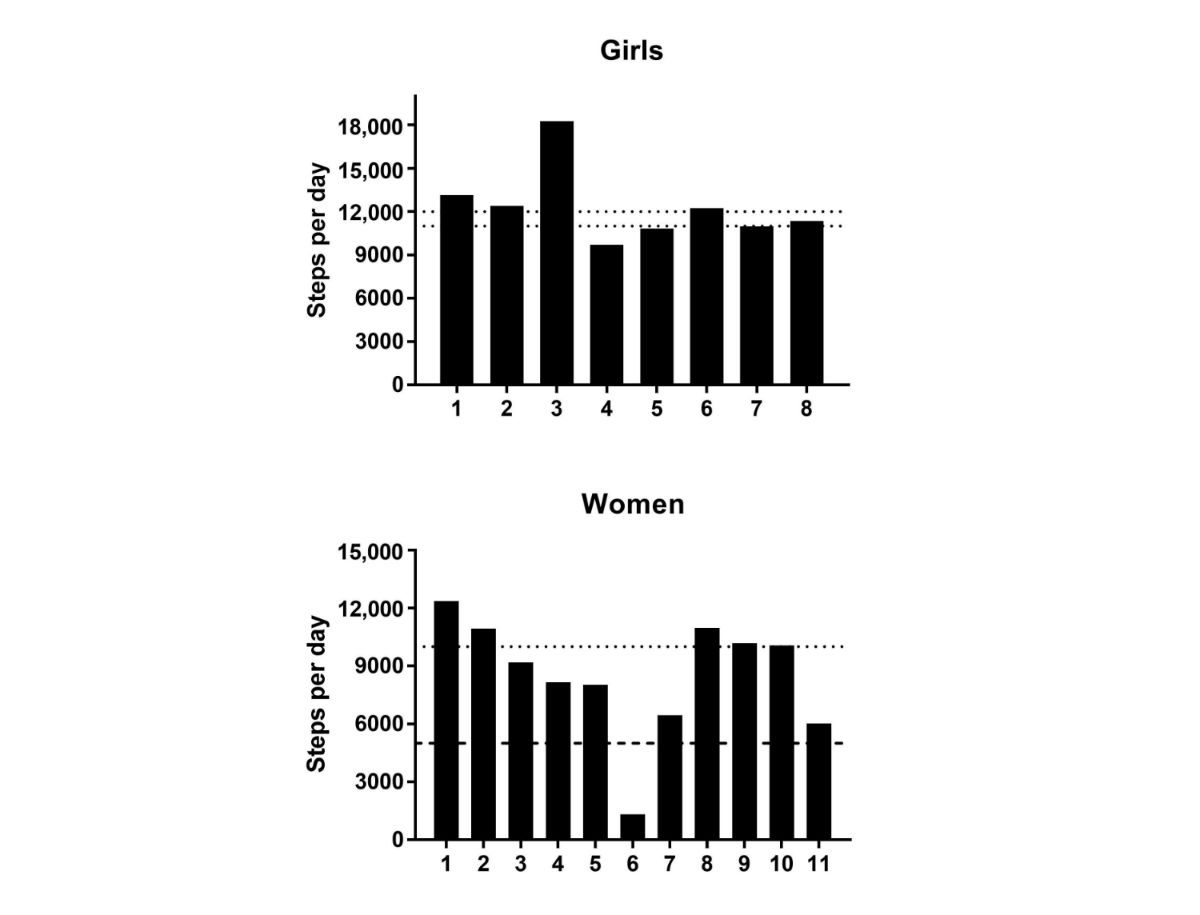
Individual average steps per day for (A) girls and (B) women. Step-defined guidelines for being physically active (•••; ≥10,000 steps/day for women; 11,000-12,000 steps/day for girls) or sedentary (- - -, ≤5000 for women) are displayed. N=8 for girls due to unavailable data from one participant’s activity monitor.

Feasibility was evaluated by examining participants’ experiences during the activity-monitoring period. Activity data are displayed in Figure 2 and Table 5. One Movband did not function properly, which prevented us from collecting these data for one girl. The girls acquired an average of 12,373.4 (SD 2617.6) steps per day (Table 5). All of the girls revealed that they were less sedentary over the past week, and when asked what they did differently, the following responses were provided: *more activity, trying to get more steps*, *you have a goal to get 10k, 12k steps*, *sitting down less*, *slept less*, *be more active*, *move more*, *be different with your movements*, and *competitive*. Of total girls, 62.5% girls (5/8) with activity data achieved 11,000 to 12,000 steps per day, which has been associated with 60 min of moderate to vigorous physical activity [34] (Figure 2). The women acquired an average of 8515.8 (SD 3076.7) steps per day (Table 5). Approximately 50% of the women revealed they were less sedentary. The women expressing a reduction in SB stated that it was a result of monitoring their activity, making an effort to walk, being consciously aware of the Movband, and consciously trying to take the stairs:

...took the long way walking home and I would check and see where I was and then go for a walk or walk up and down the stairs.

Some responses demonstrated that the women were initially making an effort to move more but did not keep up with their activity:

...first day felt like I had to get up and do something—quickly went out the window and didn't do everything I wanted to do like go to the gym.

Those failing to self-report less SB sedentary in the previous week reported hectic schedules; however, other responses suggested that they may have been more aware of their activity levels:

No—it made me more aware to walk around more.

No—I wore it, I checked it. You did 4 miles today. Go me!

Of the total women, 45.5% women (5/11) met the recommendation of 10,000 steps per day [34], whereas all, with the exception of one, exceeded the step guidelines for sedentary behaviors (≤5000 steps per day [35]) (Figure 2).

**Table 5:** Movband activity data during monitoring period.

Activity	Girls (N=8^a^) Mean (SD^b^)	Women (N=11) Mean (SD)
Steps/day	12,373.4 (2617.6)	8,515.8 (3076.7)
Moves/day	14,917.6 (3153.8)	10,269.2 (3708.8)
Miles/day	5.2 (1.3)	4.0 (2.0)

^a^Because of unavailable data from one participant’s activity monitor; low intensity activity results in the accumulation of more moves, whereas high intensity activities result in the accumulation of more miles.

^b^SD: standard deviation.

## Discussion

### Principal Findings

This study used formative assessments to (1) examine the likeability and usability of the Movband and its accompanying dashboard, (2) explore teammates as a social incentive to motivate behavior change, and (3) determine the feasibility of these tools for inclusion in an intervention to reduce SB in girls and young women. In this process, we employed a consumer-focused approach to better understand the preferences of our target population. Our findings suggest that the participants (1) liked many features of the Movband and dashboard and found the system to be user-friendly, however several dislikes and desired aspects were identified; (2) expressed an interest in favor of teammates but preferences for sex concordance and interaction strategies that allowed for private messaging were preferred; and (3) desired additional modifications related to aesthetics, functionality, and device comfort that need to be addressed before the use of this activity-monitoring system as an intervention tool to reduce SB.

Wearable technology has shown promise in improving activity patterns in youth and adults [16,17,36]. However, our understanding of the most effective intervention strategies to modify SB, as an exclusive intervention target, is insufficient, and this may be due to a lack of involvement and collaboration between populations of interest and researchers in the planning and development of behavioral programs [7,37]. Gathering a priori insight from these individuals on their preferences for program tools and strategies may assist researchers in identifying effective methods that can improve the efficacy of SB interventions. For these reasons, an understanding of the Movband and dashboard likeability and usability among our participants was a vital step in assessing their feasibility.

To our knowledge, this is the first study to use formative assessments to examine participant preferences for the Movband and dashboard in our planning and development of an SB intervention for girls and young women. Others studies have explored user preferences for Movband system in children [38] and college students [39] but not with the intent to develop an SB intervention. The study in children examined their perceptions of three commercially available activity-monitoring systems with the devices worn simultaneously and reported that the Movband system was the least “liked” among the participants [38]. The possibility that the comparisons between the commercially available systems may have prejudiced the children’s opinions of the Movband system cannot be ignored. To reduce the likelihood of this occurring in this study, participants were only eligible if they had not worn an activity monitor in the past 3 months to ensure that they were inexperienced in monitoring their activity with emerging technologies or to wash out any previous experiences with electronic self-monitoring tools. The study in adults incorporated Movband technology into a Web-based kinesiology course and received positive feedback related to its ability to encourage favorable physical activity habits among students; however, a newer model of the device (ie, model 3 as opposed to model 2) was used and the study aims were not focused on health behavior modification but rather enhancing the learning experience [39]. Taking a consumer-focused approach and segmenting our focus groups in this study allowed us to identify age-specific design and functional preferences that might influence the appeal, perceived value, and ability of this activity-monitoring system to influence SB. Although our participants found the activity-monitoring system to be user-friendly, they identified several unfavorable aesthetic features and functional limitations that may affect their engagement compliance if not addressed.

The popularity of emerging wearable technologies lies in their ability to influence health behaviors [20] through their intrinsic behavior change techniques [40]. A content analysis of the Movband activity-monitoring system demonstrated that it lacks key behavior change components, including a social support feature [38]. Social support is an important component for activity-related behavior changes [41,42], and the use of partners/team-based strategies has been associated with social support, motivation, and accountability [27,28]. In addition, studies in youth and young adults have indicated that friends can motivate activity-related behavior changes and may even improve device wear compliance [37,38]. As such, we explored the participants’ receptiveness to having an older (for girls) or younger (for women) partner. Interest was high in favor of having a teammate, as participants identified friendship, support, motivation, competition, accountably, and role modeling as potential benefits. However, the women preferred sex concordance among teammates and interaction strategies that allowed for private messaging. At present, the dashboard does not possess the capabilities to allow users to communicate with one another through messaging or chat rooms, limiting the potential use of this platform [43].

Conducting focus groups before and after the activity-monitoring period allowed us to explore participants’ experiences with the Movband activity-monitoring system in a free-living setting and assess its feasibility to decrease SB. The majority of our participants self-reported a reduction in SB during the monitoring period. Studies utilizing electronic activity–monitoring systems have been effective in decreasing SB in young adults, but researchers have explicitly instructed participants to interact with the Web-based platform and aim to achieve their daily goals, which was enhanced by the intrinsic behavior change techniques of the system (eg, motivational emails after achieving goals) [44,45]. Our participants neither received explicit instructions to alter their activity patterns nor did they have access to the dashboard outside the focus groups. Their only instruction was to wear the Movband at all times, with the exception of bathing and showering. When asked about what they did differently, the many responses indicated that both girls and women made a conscious effort to move more, suggesting that the device increased our participants’ awareness of their SB, which is supported by others [19]. The Movband data lend objective support self-reported changes in SB as all but one of the women exceeded step criteria indicative of a sedentary lifestyle (≤5000 steps/day) [35], despite less than half (45.5%) of the women achieving the recommendation of 10,000 steps/day [34]. A sedentary lifestyle step-based index has not been fully established for girls [35]; however, 62.5% of our participants achieved 11,000-12,000 steps per day, which has been associated with 60 min of moderate to vigorous physical activity [34]. The range of dashboard preferences identified by our participants during focus groups suggested that the women were more thorough during the observation period, as some of their preferences (Table 4 and Multimedia Appendix 1) indicated that their searches went beyond that of the “default platform” (Figure 1). Likewise, many of the preferred features described by our participants are readily available in the dashboard (Textbox 1) but went unnoticed indicating the need for a demonstration component to highlight the platform capabilities.

One of the unique features of this activity-monitoring system is that the dashboard was readily available for our use and is compatible with third party devices and apps (including the popular Fitbit; Textbox 1), which may be one strategy to overcome some of our participants’ aesthetic and functional preferences. However, many features available on the compatible devices (eg, prompts/cues for periods of inactivity, automated sleep, heart rate, and food logging tracking) are not inherent to the Movband system and, in addition to the interpretation of activity data (eg, equivalence of Movband steps vs Fitbit steps), may present challenges from a research standpoint. Thus, although the platform compatibility is an appealing feature of this system, identifying ways to address potential challenges related to participant engagement and data interpretation will be necessary.

### Limitations

This study is not without limitations, including the small sample size and hence the generalizability of preference findings to similar populations of girls and women. Our participants did not have access to the dashboard during the activity-monitoring period, which may have influenced their activity patterns and system preferences. In contrast, our sample was diverse in age, race, and ethnicity, with a larger proportion of minority participants than nonminority participants, which in turn is the strength of our study.

### Conclusions

In summary, our findings revealed that the Movband and dashboard are user-friendly, yet several age-specific modifications related to aesthetics and functionality require improvements and subsequent formative assessments to increase the appeal, likeability, and potential use of this activity-monitoring system as an intervention tool. Considerations for the use of this system in an SB intervention include age-specific tailoring of the dashboard and implementing platform demonstration component to ensure that participants are aware of all functional capabilities. Using team-based designs to enhance motivation and social support may encourage participants’ engagement and promote compliance in future behavioral interventions, but teams should be sex-matched and the platform should include message boards and team chat features. Interventionists who are interested in conducting efficacy studies to reduce SB in girls and young women by using wearable technologies should consider the preferences, opinions, and prior self-monitoring experiences of their target population to identify the feasibility of their intervention tools.

## Appendix

Multimedia Appendix 1 Unique responses for girls and women by visit.

## Abbreviations

BMI: body mass index
SB: sedentary behavior
SD: standard deviation
USB: Universal Serial Bus

## References

[1] Sedentary Behaviour Research Network (2012). Letter to the editor: standardized use of the terms “sedentary” and “sedentary behaviours”. Appl Physiol Nutr Metab.

[2] Carpenter K, Pereira M, Odegaard A, Jacobs D, Sternfeld B, Reis J, Gabriel K (2015). Sedentary behavior and CVD risk factors in year 25 of the CARDIA study. FASEB J.

[3] Lyden K, Keadle SK, Staudenmayer J, Braun B, Freedson PS (2015). Discrete features of sedentary behavior impact cardiometabolic risk factors. Med Sci Sports Exerc.

[4] Saunders TJ, Tremblay MS, Mathieu MÈ, Henderson M, O'Loughlin J, Tremblay A, Chaput JP, QUALITY cohort research group (2013). Associations of sedentary behavior, sedentary bouts and breaks in sedentary time with cardiometabolic risk in children with a family history of obesity. PLoS One.

[5] Seguin R, Buchner DM, Liu J, Allison M, Manini T, Wang CY, Manson JE, Messina CR, Patel MJ, Moreland L, Stefanick ML, Lacroix AZ (2014). Sedentary behavior and mortality in older women: the Women's Health Initiative. Am J Prev Med.

[6] Liao Y, Liao J, Durand CP, Dunton GF (2014). Which type of sedentary behaviour intervention is more effective at reducing body mass index in children? A meta-analytic review. Obes Rev.

[7] Altenburg TM, Kist-Van Holthe J, Chinapaw MJ (2016). Effectiveness of intervention strategies exclusively targeting reductions in children's sedentary time: a systematic review of the literature. Int J Behav Nutr Phys Act.

[8] Prince SA, Saunders TJ, Gresty K, Reid RD (2014). A comparison of the effectiveness of physical activity and sedentary behaviour interventions in reducing sedentary time in adults: a systematic review and meta-analysis of controlled trials. Obes Rev.

[9] Troiano RP, Berrigan D, Dodd KW, Mâsse LC, Tilert T, McDowell M (2008). Physical activity in the United States measured by accelerometer. Med Sci Sports Exerc.

[10] Matthews CE, Chen KY, Freedson PS, Buchowski MS, Beech BM, Pate RR, Troiano RP (2008). Amount of time spent in sedentary behaviors in the United States, 2003-2004. Am J Epidemiol.

[11] (2014). CDC.

[12] Brock DW, Thomas O, Cowan CD, Allison DB, Gaesser GA, Hunter GR (2009). Association between insufficiently physically active and the prevalence of obesity in the United States. J Phys Act Health.

[13] Hamilton MT, Hamilton DG, Zderic TW (2014). Sedentary behavior as a mediator of type 2 diabetes. Med Sport Sci.

[14] Joseph RP, Durant NH, Benitez TJ, Pekmezi DW (2014). Internet-based physical activity interventions. Am J Lifestyle Med.

[15] Sanders JP, Loveday A, Pearson N, Edwardson C, Yates T, Biddle SJ, Esliger DW (2016). Devices for self-monitoring sedentary time or physical activity: a scoping review. J Med Internet Res.

[16] Lewis ZH, Lyons EJ, Jarvis JM, Baillargeon J (2015). Using an electronic activity monitor system as an intervention modality: a systematic review. BMC Public Health.

[17] Ridgers ND, McNarry MA, Mackintosh KA (2016). Feasibility and effectiveness of using wearable activity trackers in youth: a systematic review. JMIR Mhealth Uhealth.

[18] Pina LR, Ramirez E, Griswold WG (2012). Fitbit+: a behavior-based intervention system to reduce sedentary behavior. http://ieeexplore.ieee.org/abstract/document/6240381/.

[19] Ellingson LD, Meyer JD, Cook DB (2016). Wearable technology reduces prolonged bouts of sedentary behavior. Transl J Am Coll Sport Med.

[20] Patel MS, Asch DA, Volpp KG (2015). Wearable devices as facilitators, not drivers, of health behavior change. J Am Med Assoc.

[21] Shih PC, Han K, Poole ES, Rosson MB, Carroll JM Ideals.illinois.edu.

[22] Wilson MG, Olds RS (1991). Application of the marketing mix to health promotion marketing. J Health Educ.

[23] Giles EL, Robalino S, McColl E, Sniehotta FF, Adams J (2014). The effectiveness of financial incentives for health behaviour change: systematic review and meta-analysis. PLoS One.

[24] Moller AC, Buscemi J, McFadden HG, Hedeker D, Spring B (2014). Financial motivation undermines potential enjoyment in an intensive diet and activity intervention. J Behav Med.

[25] Zhang J, Brackbill D, Yang S, Becker J, Herbert N, Centola D (2016). Support or competition? How online social networks increase physical activity: a randomized controlled trial. Prev Med Reports.

[26] Arigo D, Schumacher LM, Pinkasavage E, Butryn ML (2015). Addressing barriers to physical activity among women: a feasibility study using social networking-enabled technology. Digit Health.

[27] Arigo D (2015). Promoting physical activity among women using wearable technology and online social connectivity: a feasibility study. Health Psychol Behav Med.

[28] Prestwich A, Conner MT, Lawton RJ, Ward JK, Ayres K, McEachan RR (2012). Randomized controlled trial of collaborative implementation intentions targeting working adults' physical activity. Health Psychol.

[29] Bcm.edu.

[30] Joe.

[31] Fadel AC, Weiss PS, Meyer A, Kay C, Allensworth D, Green K, Cheung PC, Gazmararian JA (2015). Validation of physical activity measuring devices in children. Res Q Exerc Sport.

[32] Menickelli J, Sidman C, Claxton D, Grube1 D, Leonard E, Lowell S (2013). Convergent validity of an activity monitor with a research-grade accelerometer. Res Q Exerc Sport.

[33] Proquest.

[34] Tudor-Locke C, Craig CL, Beets MW, Belton S, Cardon GM, Duncan S, Hatano Y, Lubans DR, Olds TS, Raustorp A, Rowe DA, Spence JC, Tanaka S, Blair SN (2011). How many steps/day are enough? for children and adolescents. Int J Behav Nutr Phys Act.

[35] Tudor-Locke C, Craig CL, Thyfault JP, Spence JC (2013). A step-defined sedentary lifestyle index: <5000 steps/day. Appl Physiol Nutr Metab.

[36] Bond DS, Thomas JG, Raynor HA, Moon J, Sieling J, Trautvetter J, Leblond T, Wing RR (2014). B-MOBILE--a smartphone-based intervention to reduce sedentary time in overweight/obese individuals: a within-subjects experimental trial. PLoS One.

[37] Deliens T, Deforche B, De Bourdeaudhuij I, Clarys P (2015). Determinants of physical activity and sedentary behaviour in university students: a qualitative study using focus group discussions. BMC Public Health.

[38] Masteller B, Sirard J, Freedson P (2017). The Physical Activity Tracker Testing in Youth (P.A.T.T.Y.) Study: content analysis and children's perceptions. JMIR Mhealth Uhealth.

[39] Brock SJ, Wadsworth D, Hollett N, Rudisill ME (2016). Using Movband technology to support online learning: an effective approach to maximizing resources in kinesiology. Kinesiol Rev.

[40] Lyons EJ, Lewis ZH, Mayrsohn BG, Rowland JL (2014). Behavior change techniques implemented in electronic lifestyle activity monitors: a systematic content analysis. J Med Internet Res.

[41] Mendonça G, Cheng LA, Mélo EN, de Farias Júnior JC (2014). Physical activity and social support in adolescents: a systematic review. Health Educ Res.

[42] Sawka KJ, McCormack GR, Nettel-Aguirre A, Hawe P, Doyle-Baker PK (2013). Friendship networks and physical activity and sedentary behavior among youth: a systematized review. Int J Behav Nutr Phys Act.

[43] Webb TL, Joseph J, Yardley L, Michie S (2010). Using the internet to promote health behavior change: a systematic review and meta-analysis of the impact of theoretical basis, use of behavior change techniques, and mode of delivery on efficacy. J Med Internet Res.

[44] Barwais FA, Cuddihy TF (2015). Empowering sedentary adults to reduce sedentary behavior and increase physical activity levels and energy expenditure: a pilot study. Int J Environ Res Public Health.

[45] Barwais FA, Cuddihy TF, Tomson LM (2013). Physical activity, sedentary behavior and total wellness changes among sedentary adults: a 4-week randomized controlled trial. Health Qual Life Outcomes.

