# Supplementary material for: Formative Assessment: Design of a Web-Connected Sedentary Behavior Intervention for Females
Source: JMIR Hum Factors. 2017 Oct 20;4(4):e28. doi: 10.2196/humanfactors.7670 (PMC5670308; doi:10.2196/humanfactors.7670)
Supplement: Multimedia Appendix 1 [file humanfactors_v4i4e28_app1.pdf]

Appendix

**A1. Unique Responses at Visit 1 (Girls)**
Dashboard Likes
The way you can go back (in time) to see your steps
There are so many steps, moves, and miles
Like how it measures what you do everyday
It tells you how many steps in the month, year, whole
It tells you how far you are towards your goal
You can pick a date and see how many moves you have for that day
It seems like the watch has eyes and sees stuff
How it is to get around to see stuff
It tells you exactly
Like how it shows your data so you don't have to keep track of it
You can use the arrows to see how much you are exercising
Dashboard Dislikes
Boring to look at
Don’t remember the password then you can't get in
Nothing
You can get lost easily
It's pretty fine
MOVband Likes
It can count your steps
Easy to work (buttons)
I like how it's comfy
You can see how many miles you have
I like the buttons on it
How it restarts by itself every day
It's cool how it records everything
How you can see the time
How you can adjust the size of the band
How it stays on (the display)
MOVband Dislikes
I don't like the color
I don't like how the time goes straight down
It's not pretty
Nothing that I don’t like about the watch
Feels weird on my wrist because its flat; has a little extra space on the side
How you can't slide like (display like fitbit)
Nothing , really
I agree with (referring to the dislike of the color)
Not waterproof

Appendix

A2. Unique Responses at Visit 2 (Girls)

Dashboard Likes

Seeing how you passed your goals
I like how I can look at movements
See how awesome you are at moving
How it piles your steps in one big pile
I like the same things from last week
It tracks your miles, steps and you can pick which day you want to look at
I like how I had a certain goal
It's cool
You can [count] miles, steps, and moves
It is colorful
You can go as back as you like (to see your activity)
How it rates your steps (the graphs)

Dashboard Dislikes

Sometimes you don't know what to do and what to press
Boring to look at
The color was ugly
How it doesn't tell you your whole total
It's hard to believe that my steps were low
You can get lost in time
My activity didn't show up
I don't like the program

MOVband Likes

How the screen glows when you press the button
How the miles don't reset every night
It can tell time
Like how it feels on my wrist
It's black and black goes with everything
Light and sometimes you forget that you are wearing it
Comfortable to wear
How it charges
Played with it when bored in class; put on my ankle
Like how its stiff up top (top of monitor)

MOVband Dislikes

Not waterproof
It's uncomfortable, it's itchy, to sleep in too
Sometimes when you put it on it felt uncomfortable
It gets too much attention
Doesn't count the movement right when it happens
When I put it on there was still extra space, it felt weird (small wrists)
Taking it on and off every night
How it restarts steps
Tells time upside down
it didn't tell the right time
Distracts me in class
It's ugly
I forgot to take it when you step in the shower
When you press the button to go to the next thing it just glows
When I held down on the glowing button it erased the steps
Because it is not going across but up and down it is confusing (steps, moves)
I don't like that you can leave it in the bathroom (after a shower)
Sometimes you put it on backwards but can tell once I put the light on
It makes you ashy

A3. Unique Responses at Visit 1 (Women)

Dashboard Likes

Easy to navigate
Incentives and prizes
No advertisements
Very Simplistic
Shows daily, weekly, monthly
Gives you a challenge (challenge groups)
You can start where you feel comfortable (goals) as opposed to where others are starting
Have a goal and let you know where you are
Color-easy on the eyes; positive
Community and group aspect
Not only steps, shows miles
I like that there is a leaderboard
Only I can see sensitive information (in health log)

Dashboard Dislikes

Set goals to specific days; How to personalize goals
Doesn't allow you to keep track of HR, calories burned, or BP
Can't compare Mov Data with weight loss
No bluetooth capability
I don't know what a move is? (FAQ's about qualifications)
Hilarious that you can share on LinkedIn and need more social media options
Wish it could be integrated with a food journal (streak feature)
Kind of boring
Download data in CSV file
Time specific data
Connectivity Issues
Can't customize your own (website)

MOVband Likes

Long battery life
Price
Narrow and not bulky on wrist
Different colors (of bands)
Backlight at night
Comfortable
Easy to put on
Like that it shows the moves and miles
It doesn't take a lot to see what I am doing -- only 2 buttons on it
Don't have to go through a lot of steps to connect. Just plug in
Looks just like a watch
Display is always available

MOVband Dislikes

Vertical display -- want to read it left to right
Not waterproof; most movement in the shower
Like it better if it's round
More features (time, alarm clock, etc.)
Analog
Would like different color; black is masculine
Alert for when you reach a certain goal
Date
Wouldn't wear with normal clothes - only exercise clothes
Feature that lets you know you haven't met your goals
Doesn't show the steps

A4. Unique Responses at Visit 2 (Women)

Appendix

Dashboard Likes

Did I reach my goal (under/above)
Breakdown by times hour by hour
Dashboard
Tells you the % toward your goals
Fast-synced - more likely to sync often
Sync other devices to the website
Graph of what I've done
Interact with others on the website
Being able to see different breakdown (moves, steps, miles) as far as your goals
I could see why I didn't meet my goal
Neat to see day by day progress

Dashboard Dislikes

Connect to calorie tracker and get credit for calories burned - my fitnesspal (iPhone)
Can't set the goals/set for 4 miles but may want more
Being able to put in height and weight to track BMI and weight loss
Calorie thing (lack of)
Don't ask for your measurements (height and weight) and how movement impacted these measurements
Not a way to see % change from last week
Is there a way to see feedback on your goal? (Suggestion to achieve goal next week)
Could have tips to reach your goals if you didn't
Still bland

MOVband Likes

Time
Backlight
Didn't have to charge it
It is really good feedback in terms of my moves and steps
It didn't reset your miles every day so you can see how many miles per week instead of per day
It blended in
Easy to put on
Wasn't as bad wearing it every day as I thought it was going to be
Resets automatically at midnight
Not easily reset - kid friendly
Miles feature
Liked how a "move" was a little more exaggerated than a step
Liked how the rubber doesn't absorb liquid (sweat)
I didn't mind sleeping in it
People at my job thought it was a smart watch
Almost the same reported mileage as the treadmill

MOVband Dislikes

Doesn't show the date
Other ways to wear it other than wrist
Smaller band - constantly was adjusting it
Not waterproof
I wish it had a thing for steps too
I wish that it was a different style; I had it on with a dress when I went to a nice restaurant
Didn't have a timer
Stood out (physically off of the wrist)
Made me take it off for certain things/events
Just don't like things on my wrist
Clasp moves around and doesn't always do what it's supposed to
Time is vertical
I didn't like sleeping in it. It was too tight. If I loosened it then it would move around my wrist
Orientation -- round/horizontal
I wish the miles reset every day
Wear and tear for a short period of time
I wished that it was more like a bracelet
I didn't like that it didn't have Bluetooth
